# Familial clustering and risk of groin hernia in children

**DOI:** 10.1002/bjs5.8

**Published:** 2017-07-26

**Authors:** J. Burcharth, M. Pedersen, T. Bisgaard, C. B. Pedersen, J. Rosenberg

**Affiliations:** ^1^ Centre for Perioperative Optimization, Department of Surgery, Herlev Hospital University of Copenhagen Herlev Denmark; ^2^ National Centre for Register‐based Research University of Aarhus Aarhus Denmark; ^3^ Department of Surgical Gastroenterology, Hvidovre Hospital University of Copenhagen Hvidovre Denmark

## Abstract

**Background:**

The hypothesis was that groin hernias are hereditary. This study was undertaken to establish the degree of familial clustering of groin hernias on a nationwide scale.

**Methods:**

A register‐based cohort was created consisting of all children in Denmark whose parents were born in 1970 or later by the use of the Danish Civil Registration System. Within this cohort, all groin hernia operations were identified. To establish the risk estimates associated with a positive family history of groin hernia operation, information on groin hernia operations in fathers, mothers and siblings was also assessed.

**Results:**

In the cohort of 408 381 persons, a total of 4966 were operated on for groin hernia (incidence rate 2·12 per 1000 person‐years at risk). A person with a mother who had undergone surgery for a groin hernia had an increased risk of 2·89 (95 per cent c.i. 2·48 to 3·34) of having a groin hernia operation; a person with a father operated on for a groin hernia had an increased risk of 1·75 (1·58 to 1·94); and a person with a sibling operated on for a groin hernia had an increased risk of 2·54 (2·17 to 2·96). The strongest association was seen between mothers who had been operated on for groin hernia and their daughters (increased risk 6·01, 95 per cent c.i. 4·53 to 7·80), compared with the risk in girls who did not have a mother who had undergone surgery for groin hernia.

**Conclusion:**

Groin hernias are clustered in families, with the strongest relationship seen between mothers and their daughters.

## Introduction

Groin hernia surgery (inguinal and femoral) is common^1^ ^2^. It might result in persistent pain, risk of recurrence, the need for emergency surgery, sometimes with bowel resection, and a mortality rate of up to 6 per cent when incarcerated^3^, ^4^, ^5^, ^6^, ^7^. Based on studies of inherited collagen diseases, it has been hypothesized that groin hernia results from an imbalance in systemic collagen subtypes^8^, ^9^, ^10^. A positive family history of groin hernia has been associated with the development of both primary groin hernias and earlier recurrence of groin hernia after surgery^11^, ^12^, ^13^. The aspect of a positive family history of groin hernia has been investigated, although heterogeneity and small studies have limited the evidence supporting the influence of sex and inheritance^14^, ^15^, ^16^, ^17^, ^18^, ^19^.

Exact knowledge of familial accumulation of groin hernia in a nationwide cohort could provide a better understanding of the pathophysiology of this common condition. The aim of this study was to clarify mutual risk of developing groin hernia in family members and to establish whether groin hernias cluster in families.

## Methods

The study was approved by the Danish Data Protection Agency (no. 2011‐41‐6149) and the Danish National Board of Health (no. 7‐505‐29‐1765/1). A local ethics committee did not evaluate the study, as only biomedical studies require ethics committee approval according to Danish law.

The Danish Civil Registration System (CRS) was established in 1968; all people alive and living in Denmark are registered^20^. Among other variables, the CRS includes a personal identification number (CRS number), sex, date of birth and continuously updated information on vital status, registration of parents, maternal siblings and children. The CRS can therefore be used to establish family relations. The unique CRS number is used in all Danish national registers, enabling linkage between registers.

Using the CRS, a cohort was identified consisting of all Danish residents with both parents born in Denmark from 1970 onwards. Within this cohort, details of all groin hernia operations (inguinal and femoral) performed between 1 January 1977 and 31 December 2010 according to ICD‐8 (1977–1993) and ICD‐10 (1994–2010) were retrieved through the Danish National Hospital Register (NHR). The NHR commenced registration on 1 January 1977, and includes all elective and emergency hospital admissions and surgical procedures performed in Denmark. From 1994, the NHR was expanded to include outpatient and emergency department contacts^21^. The NHR serves as the basis for economic reimbursement for medical services in Denmark, so there is a vested interest by providers to enter accurate and timely information. It was not possible to discriminate between elective and emergency operations, or to separate inguinal from femoral hernia operations, in the analyses owing to register limitations.

Persons in the study population, their parents and siblings were linked to the NHR to obtain information on groin hernia operations. Individuals in the study population were classified as having had a groin hernia if they had undergone surgery for a groin hernia at least once in the study period. All included individuals were followed from birth to the first groin hernia operation, death, emigration from Denmark, or until 31 December 2010, whichever came first. For each cohort member, information was accessible regarding the person and their family members' potential operations for groin hernia, as well as the timing of these operations.

Both exposure (parents' and siblings' first groin hernia operation, if any) and outcome (persons' first groin hernia operation, if any) were defined according the procedure codes shown in *Table* 1. Date of onset was defined as the day of surgery for the first groin hernia operation.

**Table 1:** Groin hernia operation codes from ICD‐8 and ICD‐10

	Operation codes
ICD‐8	40620, 40621, 40640, 42000, 40660, 42100, 40740, 42900, 40760, 40800, 40801, 40840, 42810
ICD‐10	KJAB00, KJAB10, KJAB11, KJAB20, KJAB30, KJAB40, KJAB96, KJAB97, KJAC10, KJAC11, KJAC30, KJAC40, KJAC96, KJAC97

### Statistical analysis

The rate of groin hernia operation is reported as incidence rates (IRs) per 1000 person‐years at risk. The risk of groin hernia operation among first‐degree family members (incidence rate ratio, IRR) was estimated by log linear Poisson regression with the GENMOD procedure in SAS^®^ version 9.2 (SAS Institute, Cary, North Carolina, USA), adjusting the risk estimates for sex, age, calendar year and positive family history of groin hernia operation in a parent or sibling^22^. Age, calendar year and positive family history were treated as time‐dependent variables, with sex treated as a time‐fixed variable^23^. Age was categorized in 1‐year age levels. The risk estimates were stratified for sex, but not among siblings owing to register limitations. Calendar years 1977–1992 were categorized as one period, with 1‐year periods thereafter. Confidence intervals and *P* values were based on the likelihood ratio test^23^.

## Results

### Risk of groin hernia operation

The cohort of 408 381 (198 416 female and 209 965 male offspring) included all children born in Denmark whose parents were born in 1970 or later. A total of 4966 individuals were operated on for a groin hernia (849 females, 4117 males) during the study period. The overall incidence rate of groin hernia operation was 2·12 per 1000 person‐years at risk. Demographic data regarding surgical procedures and sex distribution have been reported in detail previously^24^. Persons with first‐degree family members who were operated on for a groin hernia had a higher IR of having surgery for a groin hernia themselves compared with that in individuals with no affected first‐degree family members. The highest IR of groin hernia operation was found among children with a mother who had undergone surgery for a groin hernia: IR 5·97 per 1000 person‐years at risk.

### Risk of groin hernia operation according to family history

*Table* 2 shows the adjusted IRRs of groin hernia operation according to the history of groin hernia operation in mother, father or sibling. Having an affected first‐degree family member carried a higher risk of being operated on for a groin hernia compared with having no affected first‐degree family member. Children whose mother had undergone surgery for a groin hernia had an almost threefold greater risk of also being operated on for a groin hernia.

**Table 2:** Adjusted incidence rate ratios of groin hernia operations associated with a history of groin hernia surgery in mother, father or sibling

	Patients operated on for groin hernia (*n* = 4966)	Incidence rate*	IRR of being operated on for groin hernia†	*P* ‡
Relative operated on for groin hernia				
Mother				
Yes	163	5·97	2·89 (2·48, 3·34)	< 0·001
No	4803	2·08	1·00 (reference)	
Father				
Yes	400	3·63	1·75 (1·58, 1·94)	< 0·001
No	4566	2·05	1·00 (reference)	
Sibling				
Yes	166	5·66	2·54 (2·17, 2·96)	< 0·001
No	4800	2·08	1·00 (reference)	

Values in parentheses are 95 per cent confidence intervals.

* Rate of hernia per 1000 person‐years at risk.

† Estimates of incidence rate ratio (IRR) adjusted for calendar year, age and its interaction with sex and history of groin hernia operation in mother, father or sibling.

‡ Likelihood ratio test.

*Post hoc* analyses, stratifying for the effect of parental history of groin hernia by sex, were performed. The effect of hernia in a father did not differ significantly between the two sexes (*P* = 0·880). The effect of hernia in a mother differed significantly by sex (*P* < 0·001): male offspring had a 2·22 (95 per cent c.i. 1·82 to 2·67) fold increased risk associated with a history of hernia in a mother, compared with a 6·01 (4·53 to 7·80) fold increased risk in female offspring. Children with a sibling who had been operated on for a groin hernia had an increased risk of having surgery for a groin hernia (IRR 2·54, 95 per cent c.i. 2·17 to 2·96) (*Table* 2).

## Discussion

This nationwide cohort study demonstrates that a history of groin hernia operation in a first‐degree family member significantly increases an individual's own risk of being operated on for a groin hernia. The highest risk was found among female children of mothers who had undergone surgery for a groin hernia, with a sixfold increased risk compared with that female offspring whose mother had not been operated on for hernia. The study supports the hypothesis that groin hernias cluster in families.

Inguinal hernias constitute more than 95 per cent of all groin hernias, and the rate of groin hernia surgery in both sexes increases notably over the age of 40 years^2^ ^25^. The vast majority of groin hernias in children and adolescents are indirect inguinal hernias^2^ ^26^. The age span of patients in the present study (0–20 years) meant that the majority of groin hernias were indirect inguinal, and it must therefore be assumed that the observed hereditary connection mostly represents characteristics of indirect inguinal hernias. As the study population will be subject to longer follow‐up, more will undergo surgery for groin hernia, probably strengthening the estimates.

Others have investigated the effect of a family history of groin hernia and possible inheritance patterns of groin hernias^19^. All except one^16^ focused on indirect inguinal hernias. Regarding inheritance patterns, the results have been heterogeneous, implying multifactorial threshold inheritance^27^, sex (male)‐specific dominant inheritance^17^, polygenic inheritance^28^ and autosomal dominant inheritance^14^ ^18^, ^29^. Although many of these studies have differed markedly in both methodology and size^11^, ^12^, ^13^ ^29^, ^30^, ^31^, the general finding was that a positive family history of inguinal hernia was considered a significant risk factor for the development of primary groin hernia. Two studies^27^ ^28^ investigating the effect of a positive family history of inguinal hernia among children found that girls who had sisters with an inguinal hernia had an increased relative risk of 7–30 of developing an inguinal hernia themselves. Several limitations exist in the literature, such as the lack of a clear definition of a positive family history^11^, ^12^, ^13^ ^29^ or failure to state whether data were based on clinical examination or surgical procedure^12^ ^28^, with potential observer bias. The finding of a significant correlation between groin hernia operations involving mother and daughter in the present study might indicate a complex sex‐linked inheritance without full penetrance.

Some reservations are needed when interpreting the present results. The main limitation of the study is its focus on the incidence of childhood hernia; with the current database it is not possible to identify a genetic association of hernia in adults. It cannot be excluded that parents operated on for groin hernia may pay more attention to the possibility of hernia in their children than parents who have not had such surgery themselves. Increased attention from parents towards a possible groin hernia in a child, however, would most likely only accelerate a diagnosis that otherwise would have been made later. The reason why maternal inheritance to female children is the strongest is not clear. The strengths of this study lie in the population‐ and incidence‐based cohort data being related to surgical data, eliminating clinical observer bias, and the use of adjusted risk estimates.

A genetic component of groin hernias seems likely to exist, providing a basis for further work to investigate detailed inheritance mechanisms. Even though it is not possible to draw conclusions regarding the exact inheritance pattern of groin hernia, the present results, showing a strong association between mothers and daughters, imply some form of complex sex‐linked inheritance.
